# What makes older adults feel good?

**DOI:** 10.1007/s40520-023-02387-x

**Published:** 2023-03-20

**Authors:** Anna Nivestam, Albert Westergren, Maria Haak

**Affiliations:** Department of Nursing and Health Sciences, Faculty of Health Sciences, Kristianstad University, SE-291 88, Kristianstad, Sweden

**Keywords:** Health, Occupation, Preventive home visit, Social participation, Well-being

## Abstract

**Background and aim:**

To inform health promotion interventions, there is a need for large studies focusing specifically on what makes older adults feel good, from their own perspective. The aim was to explore older adults’ views of what makes them feel good in relation to their different characteristics.

**Methods:**

A qualitative and quantitative study design was used. Independently living people (*n* = 1212, mean age 78.85) answered the open-ended question, ‘What makes you feel good?’ during preventive home visits. Following inductive and summative content analysis, data was deductively sorted, based on *The Canadian model of occupational performance and engagement*, into the categories *leisure*, *productivity*, and *self-care*. Group comparisons were made between: men/women; having a partner/being single; and those with bad/good subjective health.

**Results:**

In total, 3117 notes were reported about what makes older adults feel good. Leisure activities were the most frequently reported (2501 times), for example social participation, physical activities, and cultural activities. Thereafter, productivity activities (565 times) such as gardening activities and activities in relation to one’s home were most frequently reported. Activities relating to self-care (51 times) were seldom reported. There were significant differences between men and women, having a partner and being single, and those in bad and good health, as regards the activities they reported as making them feel good.

**Discussion and conclusions:**

To enable older adults to feel good, health promotion interventions can create opportunities for social participation and physical activities which suit older adults’ needs. Such interventions should be adapted to different groups.

## Background

There is a trend of decreased well-being in older age, due to increased risk of diseases [1]. However, maintaining well-being in older age can have a positive impact on health and may lead to increased survival [1]. Therefore, it is important to enable the performance of activities which make older adults feel good and thereby maintain or increase their well-being. Accordingly, it is important to explore what makes older adults feel good to give support which promotes well-being in older age.

Well-being is an ambiguous concept to define. The World Health Organization (WHO) has recently defined well-being as “a positive state experienced by individuals and societies” (2, p. 10). Moreover, WHO emphasizes that well-being focuses on quality of life and a person’s ability to contribute to the world with a sense of meaning and purpose [2]. In the present study, well-being is viewed as emotional well-being that includes hedonic traditions of happiness and experiences of pleasant emotions, and it includes the experience of general life satisfaction [3]. Based on the experience of pleasant emotions associated with well-being, this study takes its starting point in well-being as an experience of feeling good, based on the person’s subjective experience.

One way of maintaining well-being among older adults is, according to the literature, to offer preventive home visits [4]. A preventive home visit is an intervention given to older adults that tends to be focused on identifying risk factors and problems in the older population [5–8], and that provides advice and support [9]. However, recent research shows that the focus can be turned towards positive aspects of health during the visit and concentrate on the peoples’ assets as well [10]. Thus, during an intervention such as a preventive home visit, focus could be on maintaining well-being in older age by giving adequate support. However, to give adequate support to older adults, more knowledge is needed about what activities make older adults feel good.

Occupational science has contributed to a better understanding of activities which can influence peoples’ well-being [11]. One model used in practice to identify meaningful activities for the person is *The Canadian model of occupational performance and engagement* (CMOP-E) [12, 13]. The CMOP-E provides a theoretical framework which is intended to help professionals highlight what is known and what needs to be known about peoples’ occupational life. With the help of the framework, professionals can take decisions that can help people to find strategies to successfully perform a desired activity. According to the CMOP-E, occupations take place in a context as a result of a dynamic interaction between three dimensions: the person, the occupation and the environment. First, placed at the center of the model is the person and the ‘essence of the self’, i.e., the sense of meaning and purpose, that is experienced by the person in a given environment. In addition, the person has different performance components which can have an impact on their occupational performance: cognitive (e.g., memory, insights, intellect), affective (e.g., emotions, moods, coping skills), and physical (e.g., movement, coordination, physical illness). Second, occupation is the key component in the model and focuses on meaningful activities significant in peoples’ lives, activities which take time and energy and are shaped by society and culture. In turn, occupation is everything people do to occupy themselves divided into three areas *self-care* (e.g., bathing, dressing, feeding), *productivity* (e.g., volunteering, paid job, homemaking), and *leisure* (e.g., hobbies, games, sports). The third and final dimension of the CMOP-E model is the environment, which can enable or obstruct occupational performance. The environment is divided into four aspects: physical (e.g., built, or natural aspects from micro indoor features to macro features related to general accessibility in society); social (e.g., relationships among people in society); cultural (e.g., extending from micro family rituals to macro norms and attitudes in society); and institutional (e.g., policies, organizational structures, social determinants of health). Hence, to enable meaningful activities, the person, the occupation and the environment all have to be taken into consideration. In this study all types of occupation and their performance are described as kinds of activity, with ‘activity’ meaning doing something which makes you feel good.

A diverse range of activities have been recognized as factors contributing to well-being among older adults. Research has highlighted activities such as being with grandchildren, reading and being in the garden as factors contributing to well-being in older age [14]. In addition, research shows that social activities such as volunteering [15], and spending time on leisure activities have positive impacts on older adults’ well-being [16–18]. However, many older adults spend time indoors and alone [19, 20], and with increased age, the time spent on passive activities indoors, such as watching television, and relaxing, increase dramatically [21]. In addition, individual mobility difficulties such as walking difficulties and weakness as well as environmental aspects of accessibility and commuting difficulties can affect people’s ability to engage in activities [22]. Thus, previous research indicates that both individual support and environmental actions are needed to support older adults in doing activities which make them feel good. Therefore, to inform policymakers and health professionals, knowledge is needed, from older adults’ own perspective, about what activities make them feel good.

To get insights into what activities make older adults feel good, their own views must be explored. In research, terms such as “subjective well-being” [23], “life satisfaction” [16], and “quality of life” [24, 25] are used to emphasize the experience of feeling good. However, we would argue that in daily conversations these terms are not often used; instead the term “feeling good” is more commonly used. The question “What makes you feel good?” could be asked during a preventive home visit, and based on the answer, support and advice could be provided to facilitate the older adult’s well-being. In addition, to be able to create a society which enables well-being it is of interest to know what makes older adults feel good. Therefore, to give adequate support and take societal decisions which are relevant to older adults and reflect their values, older adults’ perspective is essential. To get closer to older adults’ perspective it is of interest to explore the term “feel good” and not ask them about the term “well-being”. The aim of this study was twofold. First, to explore older adults’ own view of what makes them feel good. Second, to explore which activities are frequently reported in relation to different characteristics, i.e., men/women, having partner/being single, and bad/good subjective health.

## Methods

### Design

Qualitative and quantitative research designs were used.

### Context

Research about preventive home visits has been conducted in countries all over the world, such as Canada [26], Japan [27], Norway [28], and Sweden [29]. This study was conducted in Sweden within a research and collaboration project named ‘Preventive home visits to seniors’ (Pre-H) [10]. Within Pre-H, participant’s ≥ 77 years old without home care are offered one preventive home visit with the purpose of promoting health and preventing ill health through a dialog based on a structured questionnaire. The visit is conducted by a visitor (e.g., nurse, assistant nurse, district nurse) who is working in the municipality’s health care organization. Older adults are invited by mail or a telephone call. The visit is free of charge, takes place in the older adult’s own home and lasts for approximately two hours. The structured questionnaire includes multiple questions about, for example, physical health, mental health, housing, and nutrition. Open-ended questions are included in the questionnaire and the one used in this study is “What makes you feel good?”.

### Sample

Participants to be included in this study were drawn from the Pre-H’s register. Consecutively from October 2018 to February 2020 all who answered the question, “What makes you feel good?” were included in the study. Older adults (*n* = 1212) from seven municipalities participated. Sample characteristics are provided in Table 1.

**Table 1:** Sample characteristics

Characteristics	*n* = 1212
Age mean (SD)	78.85 (1.84)
Women *n* (%)	668 (55.4)
Men *n* (%)	538 (44.6)
Having a partner *n* (%)	799 (66.0)
Single *n* (%)	411 (34.0)
Subjective health *n* (%)	
Good	892 (73.6)
Bad	319 (26.4)

### Data collection

The question in focus for this study was part of the set of pre-determined questions asked during preventive home visits to older adults. The visitor asked the open-ended question, and the participant could freely respond. Then, the visitor wrote down the answer in a digital support system during the preventive home visit. Participants were free to answer using their own words and there was no limitation on the number of aspects reported. During the preventive home visit, other characteristics about the participants are also registered in the digital support system. The characteristics described in Table 1 were extracted to be included in this study. The answers to the question about subjective health were dichotomized into good (excellent, very good, good) and bad (fair, bad) health.

### Analysis

The analytical approach for summative content analysis described by Hsieh and Shannon [30] was used. NVivo software was used to keep track of the material. To start with, all notes taken as answers to the question “What makes you feel good?” documented in the digital support system were given a code by the first author. In most cases, the code was labeled the same as the notes, for example the note ‘swimming’ was coded as ‘swimming’ and the note ‘children’ was coded into ‘children’. Thereafter, the codes were grouped into sub-categories (e.g., social participation, physical activities, and cultural activities). Notes recorded less than ten times and not belonging to any sub-category were excluded, due to that these single notes did not add any important information to the results. This first part of the analysis was thereafter verified by the co-authors, and agreement about the coding and sub-categories was reached. Thereafter, the frequencies of the reported notes within the sub-categories were counted. Then, the sub-categories that in total had notes which were most frequently mentioned, > 100 times, were imported in SPSS (IBM SPSS statistics software version 27) as items and given the code number, 0 (when the person has not mentioned the item) or 1 (when the person has mentioned the item). The rational, for including sub-categories with > 100 notes, was to be able to draw robust statistical conclusions. Next, chi-square tests were used to compare every item between: men and women, partner and single, and bad and good health. The level of statistical significance was set to p-value < 0.05.

As a final step in the content analysis, the sub-categories from the inductive qualitative analysis were deductively sorted according to the three occupational performance categories described in the CMOP-E model: *leisure* (e.g., hobbies, games, sports), *productivity* (e.g., volunteering, paid job, homemaking), and *self-care* (e.g., bathing, dressing, feeding) [12]. This was done by the first author and then verified by the co-authors. The categories (i.e., leisure activities, productivity activities, and self-care activities) are not mutually exclusive, neither the sub-categories above, and when doubts regarding the sorting of the data arose, these were discussed between the authors and decisions regarding the sorting were made. See Table 2 for an illustration of the categories, sub-categories, and codes/notes. All authors discussed and agreed upon the results.

**Table 2:** Illustration of category, sub-category, and code/note

Category	Sub-category	Code/note
Leisure	Social participation	Associations, choir, church, children, grandchildren
	Physical activities	Swimming, golf, bowling, walking
Productivity	Gardening activities	Gardening, flowers, gardening work
	Activities related to one’s home	Home, being at home, the house
Self-care	One’s own health	Being healthy, healthy, health

### Ethical considerations

The study was conducted in accordance with the Declaration of Helsinki [31] and approved by The Ethical Review Board, Lund, Sweden (reference number 2018/849 and 2020–02343). Informed consent was obtained from the participants before answers were entered in the register. Data transferred to the researcher were anonymous.

## Results

The number of notes generated by the 1212 participants varied between one and ten notes per person. In total, 3117 notes were reported and sorted into 17 sub-categories. Ten sub-categories were sorted into the category *leisure*, five sub-categories into the category *productivity*, and two sub-categories into *self-care* (see Table 3). The activities most frequently reported as making older adults feel good were those related to the leisure category, while activities directly related to self-care were seldom reported. Sorted into the leisure category were the following sub-categories, in descending order, *social participation (29.8%)*, *physical activities (17.6%)*, *and cultural activities (10.9%)*. Thereafter, activities related to the category productivity were the next most frequently reported, namely *gardening activities* (7.5%) and *activities in relation to one’s home* (5.9%). Activities related to the category self-care were rarely mentioned in the notes. A few notes were taken related to *one’s own health* (0.9%) and there were some notes about *eating and drinking* (0.8%) as activities which made participants feel good.

**Table 3:** The three categories leisure, productivity, and self-care with their 17 sub-categories, number of times each sub-category was reported and examples of their content

Category	Sub-category *n* = 17	Reported number of times *n* = 3117	Examples of content
Leisure	Social participation	930 times (29.8%)	Being with children, grandchildren, friends, neighbors. Participating in associations, or organizations
	Physical activities	549 times (17.6%)	Walking, cycling, dancing, golf, bowling, boules, circuit training, strength training at a gym, badminton, pistol shooting, yoga, Pilates, water gym, gymnastics, physical training for seniors, Nordic walking, water polo, rehab, group training, swimming, curling, orienteering
	Cultural activities	339 times (10.9%)	Reading, music, sports entertainment, television, film, quizzes, concerts, art, radio, theater, and museum
	Leisure accommodation and travel	169 times (5.4%)	Taking different trips both in Sweden and abroad, by bus or just driving in the car. Being in their summer house, caravan, or motorhome
	Craft and creative activities	155 times (5.0%)	Needlework (weaving, sewing, knitting, crocheting, embroidery), photography, ceramics, metalwork, painting, handicrafts, writing, carpentry, and technology gadgets
	Activities related to animals	112 times (3.6%)	Dogs, cats, birds, chickens, sheep, horses, bees, pigs and cows
	Being in nature	106 times (3.4%)	Being outdoors close to nature, for example the sea, a lake, the forest, or the mountains. Hunting, fishing, mushroom- and berry-picking
	Pastime activities	91 times (2.9%)	Crosswords, genealogy, puzzles, and stamp collection
	Activities related to motor vehicles	33 times (1.1%)	Motorcycles, vintage cars, renovation of cars and boats
	Activities at the computer	17 times (0.6%)	Playing games, surfing on the web, and social media
Productivity	Gardening activities	235 times (7.5%)	Being in the garden, doing activities related to the garden and taking care of flowers
	Activities related to one’s home	184 times (5.9%)	Taking care of the home, renovation and just being at home
	Working	63 times (2.0%)	Having your own business, driving trucks, being a trustee, language teaching, cleaning, being a craftsman, working in the construction industry, taking care of the housing association, doing forestry- and farm work
	Cooking and baking	57 times (1.8%)	Cooking and baking
	Helping someone else	26 times (0.8%)	Helping someone feel good with for example charity or helping their children with different activities
Self-care	One’s own health	27 times (0.9%)	Being healthy, being able to be active and having the functional ability needed to manage daily life and be independent
	Eating and drinking	24 times (0.8%)	Eating and drinking

An overview of the nine activities noted > 100 times which make older adults feel good reported in relation to sex, partner/single and subjective health are presented in Table 4. There were significant differences in what makes older adults feel good in relation to sex. Significantly more men reported *being in nature* (71.8%) and *leisure accommodation and travel* (53.1%) than women (28.2%, 46.9%, respectively). Women more often reported *social activities* (63.2%) and *craft and creative activities* (74.2%) than men (36.8%, 25.8%, respectively). Moreover, there were significant differences in what makes older adults feel good in relation to having a partner or being single. Significantly more who had a partner reported *gardening activities* (76.1%), *activities related to one’s home* (78.2%) and *leisure accommodation and travel* (77.5%), *social participation* (61.0%) and *cultural activities* (58.0%) than those who were single (23.0%, 21.8%, 22.5%, 39.0%, and 42.0%, respectively). Finally, there were significant differences in what makes older adults feel good in relation to health status. Significantly more people in good health reported *social participation* (76.2%), *physical activities* (80.0%), *gardening activities* (81.2%), *leisure accommodation and travel* (87.0%) and *activities related to animals* (63.0%) than those with bad health (23.8%, 20.0%, 18.8%, 13.0% and 37.0%, respectively).

**Table 4:** The nine activities noted > 100 times which made older adults feel good reported in relation to sex, partner/single and subjective health (*n* = 1212)

	Sex		*P* value	Partner/single		*P* value	Subjective health		*P* value
	Women (*n* = 668)	Men (*n* = 538)		Having partner (*n* = 799)	Single (*n* = 411)		Good (*n* = 892)	Bad (*n* = 319)	
Social participation *n* (%)	412 (63.2)	240 (36.8)	< 0.001*	399 (61.0)	255 (39.0)	< 0.001*	499 (76.2)	156 (23.8)	0.030*
Physical activities *n* (%)	242 (56.5)	186 (43.5)	0.563	283 (65.8)	147 (34.2)	0.905	344 (80.0)	86 (20.0)	< 0.001*
Cultural activities *n* (%)	152 (55.7)	121 (44.3)	0.924	159 (58.0)	115 (42.0)	0.001*	205 (74.5)	70 (25.5)	0.704
Gardening activities *n* (%)	125 (52.5)	113 (47.5)	0.314	181 (76.1)	57 (23.0)	< 0.001*	194 (81.2)	45 (18.8)	0.003*
Activities related to one’s home *n* (%)	122 (59.5)	83 (40.5)	0.196	161 (78.2)	45 (21.8)	< 0.001*	159 (76.8)	48 (23.2)	0.258
Leisure accommodation and travel *n* (%)	75 (46.9)	85 (53.1)	0.019*	124 (77.5)	36 (22.5)	0.001*	140 (87.0)	21 (13.0)	< 0.001*
Craft and creative activities *n* (%)	95 (74.2)	33 (25.8)	< 0.001*	85 (66.9)	42 (33.1)	0.822	94 (73.4)	34 (26.6)	0.952
Activities related to animals *n* (%)	57 (52.8)	51 (47.2)	0.562	71 (65.7)	37 (34.3)	0.946	68 (63.0)	40 (37.0)	0.008*
Being in nature *n* (%)	33 (28.2)	84 (71.8)	< 0.001*	83 (70.9)	34 (29.1)	0.238	89 (76.1)	28 (23.9)	0.533

A *χ*^2^ test was used to compare items

*p value < 0.05

## Discussion

This study explored 1212 older adults’ own views of what makes them feel good in relation to their different characteristics. We found that responses could be grouped into 17 different sub-categories relating to ‘What makes you feel good?’ These 17 sub-categories were deductively sorted into the categories *leisure* (including 10 sub-categories), *productivity* (including five sub-categories), and *self-care* (including two sub-categories). Activities related to *being in nature*, and *leisure accommodation and travel* dominated among men. Among women, *social participation*, and *craft and creative activities* were the most common. Moreover, older adults with a partner reported the following more often than singles: *gardening activities*, *activities related to one’s home*, *leisure accommodation and travel*, *social participation*, and *cultural activities*. Finally, compared to those with bad health, older adults in good health more often reported *social participation*, *physical activities*, *gardening activities*, *leisure accommodation and travel*, and *activities related to animals*.

The activities most commonly reported as activities which make older adults feel good are *leisure* activities, thereafter *productivity*, and finally activities relating to *self-care*. The results showed that 80% of the notes related to leisure activities, 18% to productivity and 2% to self-care. Previous research shows that during a day, older adults spend most of their time at home and alone [19, 20], and many of the activities performed during a day focus on self-care such as eating, and sleeping [32]. However, when asked a question about what makes you feel good, it might be that the “taken for granted” activities are less likely to be reported. Thus, the low reporting of self-care activities does not mean that such activities do not make older adults feel good.

Cultural activities such as reading, music and sport entertainment were popular activities that made older adults feel good, and these activities can be performed at home. However, many of the reported activities require mobility outside one’s home. For example, most of the physical activities noted such as not only walking, cycling and golf, but also leisure accommodation and travel, and being in nature require mobility. In addition, older adults frequently reported that social participation made them feel good, and this usually also requires mobility outside one’s home. Hence, it is crucial to stimulate older adults to keep on doing activities outside their homes and enable their mobility to increase the opportunity to perform activities which make them feel good.

Social participation was the most frequently reported activity which makes older adults feel good. The present results showed that activities relating to social participation were reported 930 times. Previous research has shown similar results [14] and that social participation is important for good health in older age [33]. However, our results showed that social participation was more frequently reported among women, people with a partner and those in good health. This is in line with previous research showing that those in good health [34] and women [35] are more socially active. When it comes to supporting social participation, it is important to consider the diverse preferences of the different sexes, as well as civil and health status. Nevertheless, due to the evident health benefits of social participation [33, 36], it is important to support social participation among all. There may be other reasons why fewer men, singles and people in bad health reported activities in relation to social participation. In addition, it is worth noting that many of the other activities reported, for example, physical activities and cultural activities, can involve social aspects as well. Also, the walk or the gym class might be carried out with others. Thus, it is important to reflect upon different dimensions of social participation which suit older adults’ needs when individual support is given and societal decisions are taken, aiming at supporting social participation.

To be able to perform activities which make older adults feel good the three dimensions of the CMOP-E model have to be taken into consideration: the person, the occupation and the environment [12]. The present study highlights a diversity of activities or occupations which make older adults feel good in relation to all three occupational performance categories described in the CMOP-E (leisure, productivity, and self-care). However, some of the activities which make older adults feel good might be challenging to perform when the person experiences a decline in their performance component, such as memory loss or physical illness. To compensate for these challenges society has an important role in creating a supportive environment that enables older adults to continue to enjoy activities which make them feel good. For example, society can consider older adults’ ability to access nature, social activities, and physical activities and can create opportunities for gardening activities. It is important to consider all four aspects of the environment stressed in the CMOP-E model, physical, social, cultural, and institutional. It is also important to consider the physical accessibility to an activity but also to support cultural aspects of the environment such as norms and attitudes towards older adults’ ability and permission to take part in social activities. By creating a society that fits the diversity of older adults’ preferences and needs, well-being in older age will probably increase.

Next, we turn to methodological considerations worth discussing. Approaching the material using both qualitative and quantitative analysis is a strength, which shows a deeper and broader picture of what makes older adults feel good. In addition to this, we first analyzed the material inductively and then deductively; this approach enabled the material to be understood with the help of theory, which permitted us to move from descriptions to explanations of phenomena. The use of theory also increases the transferability of the results. Another strength of the analysis was that it was made independently and then verified by co-authors. This approach minimized the misinterpretation effects caused by just one reviewer. However, regarding the coding of the various categories, some activities have the potential to be categorized into more than one category. As an example, “Being with children, grandchildren” is listed as a leisure activity—but could also count as productivity. And our interpretation may be different from that of the respondents (e.g., gardening is coded as productive—but some respondents may view it more as leisure). To solve this dilemma, additional questions must be asked to respondents so that they can be given the chance to clarify how they view these activities (as leisurely, productive, or self-care). In addition, a single question was used to assess what makes participants feel good. Unfortunately, there were not any probing questions that were asked in addition to their answer, or questions that allowed for participants to expand on their responses (“What did you mean by that?,” or “What activities does that entail?,” or “Why does that make you feel good?”). It might be that such questions were asked, but not recorded in the register. To get more in-depth insights, probing questions would be preferable to add to the battery of questions asked during preventive home visits. Another potential limitation in the study was that the notes recorded were just short words, which might increase the risk of misinterpretation of the underlying meaning of the words. Concerning the sample included in the study, the number of participants included was high (*n* = 1212), which gave a broad and varied representation of the population. However, the sample seemed to be relatively healthy, 73.6% in good health and with a mean age of 78.85 (SD 1.84). And the present result does preferably represent older adults living in their own home without home care in this age span. It is difficult to say anything about whether the activities presented in the results are transferable to an older population than that represented in the sample, or to people with ill health. As an example, Douma et al. [14] showed that older adults aged 85 years or above did not report walking and cycling and holiday/traveling as frequently as younger age-groups. This indicates that other types of activities might make older adults over 85 and with ill health feel good. Unfortunately, the present sample is homogeneous when it comes to age, and therefore it is not possible to make comparisons between different age-groups. One could, however, believe that there most likely could be differences in activities listed by older adults over 85 years compared to those 77 years old. In addition, it could be interesting to assess more demographic characteristics (ethnicity, employment status, education, income, health diagnoses, number of children, acting as caregiver, etc.), which could be incorporated into the analysis as they may have significant relationships with what makes older adults feel good.

## Conclusions

Activities which make older adults feel good, from their own perspective, were mainly *leisure* activities, then *productivity* activities, and finally activities related to *self-care*. The leisure activities most frequently mentioned were social participation (e.g., being with grandchildren), and physical activities (e.g., going for a walk). Thus, these could be interpreted as the most important activities to perform to feel good in older age. To successfully support older adults in performing activities which make them feel good the CMOP-E model can be helpful. Taking into consideration the three dimensions of the CMOP-E model, the person, the occupation, and the environment can enable older adults to perform activities which make them feel good. By considering the person and the desired activity, health professionals can support the older adults in performing activities which make them feel good. Moreover, societal decisions can be taken by creating an environment that enables older adults to perform their desired activities.

## Data Availability

The data materials used in the current study are not publicly available because the participants were informed, through the informed consent, that the data would be kept confidential. But upon reasonable request, the anonymized data may be obtained from the corresponding author.
